# Shinyscreen: mass spectrometry data inspection and quality checking utility

**DOI:** 10.1186/s13321-025-01044-x

**Published:** 2025-06-20

**Authors:** Todor Kondić, Anjana Elapavalore, Jessy Krier, Adelene Lai, Hiba Mohammed Taha, Mira Narayanan, Emma L. Schymanski

**Affiliations:** 1 Luxembourg Centre for Systems Biomedicine (LCSB), University of Luxembourg, 6 Avenue du Swing, 4367 Belvaux, Luxembourg; 2 Institute for Inorganic and Analytical Chemistry, Friedrich-Schiller University, Lessing Strasse 8, 07743 Jena, Germany

**Keywords:** High resolution mass spectrometry, Shiny, Spectral data analysis, Data processing, Visualisation, MetFrag, Compound identification, Non-targeted analysis, Docker

## Abstract

Shinyscreen is an R package and Shiny-based web application designed for the exploration, visualization, and quality assessment of raw data from high resolution mass spectrometry instruments. Its versatile list-based approach supports the curation of data starting from either known or “suspected” compounds (compound list-based screening) or detected masses (mass list-based screening), making it adaptable to diverse analytical needs (target, suspect or non-target screening). Shinyscreen can be operated in multiple modes, including as an R package, an interactive command-line tool, a self-documented web GUI, or a network-deployable service. Shinyscreen has been applied in environmental research, database enrichment, and educational initiatives, showcasing its broad utility. Shinyscreen is available in GitLab (https://gitlab.com/uniluxembourg/lcsb/eci/shinyscreen) under the Apache License 2.0. The repository contains detailed instructions for deployment and use. Additionally, a pre-configured Docker image, designed for seamless installation and operation is available, with instructions also provided in the main repository. **Scientific Contribution**: Shinyscreen is a fully open source prescreening application to assist analysts in the high throughput quality control of the thousands of peaks detected in high resolution mass spectrometry experiments. As a vendor-independent, cross operating system application it covers an important niche in open mass spectrometry workflows. Shinyscreen supports quality control of data for further identification or upload of spectra to public data resources, as well as teaching efforts to educate students on the importance of data quality control and rigorous identification methods.

## Introduction

In recent years, high resolution mass spectrometry (HRMS) has become an indispensable analytical tool across various disciplines including metabolomics, proteomics, environmental chemistry, forensics and drug discovery [1]. HRMS enables researchers to analyze complex mixtures, identify compounds, and quantify their presence with high selectivity and sensitivity [2]. Typical HRMS workflows often follow one of several paths, tailored to specific analytical goals. Targeted analysis focuses on quantifying a predefined set of known compounds. Suspect screening investigates compounds on a curated list for identification, while non-targeted analysis (NTA) aims to uncover unknown compounds within a sample, requiring advanced analytical instruments and data processing software to process and annotate spectral data [3].

The increasing sophistication of MS instrumentation, coupled with the growing size and complexity of MS datasets, poses significant challenges in data processing, visualization, and interpretation [4]. To address these challenges, researchers rely on specialized software solutions tailored for MS data analysis. On one hand, device manufacturers usually provide software for data inspection and visualisation such as Xcalibur [5] for Orbitrap instruments. However, these tools are often constrained by restrictive licensing, limited compatibility with non-native platforms, and have a narrow focus on specific instrument models [6]. Consequently, they fail to meet the needs of users working with diverse MS platforms or seeking greater customization and transparency in their workflows [7].

On the opposite end of the spectrum, the open source community has developed robust software approaches to address the challenges associated with MS data analysis. These range from collaborative browser-based solutions such as GNPS Dashboard and GNPS Dataset Explorer [8] to several solutions offered in various open programming languages including python, Julia and R. In the programming language R, the main focus of this article, these include powerful, but low-level R packages such as mzR [9] and the more modern MSnbase [10, 11], which offer great flexibility in analysis of MS data and can be used for a first-look data exploration and prescreening, but require some programming expertise to fully utilize their functionality. On the other end of the scale, there are options such as xcms [12], RforMassSpectrometry [13] and patRoon [14], which provide comprehensive solutions for non-targeted metabolomics and environmental workflows. These approaches are very powerful, but often require significant computational resources, extended configuration time, and expertise in navigating their complex functionality. Further extending the toolkit, MetFrag [15] and RMassBank [16, 17] are both methods to support compound identification. MetFrag focuses on in silico fragmentation to match experimental data to candidate compounds, aiding in compound annotation during suspect and non-target screening workflows [18]. RMassBank facilitates the creation and management of high-quality spectral libraries, for higher confident compound annotation [3], but was originally designed to process single-injection pure standards and not mixtures. Despite their utility, these tools often serve specialized needs within broader HRMS workflows, such that integrating them effectively can be challenging for users without extensive computational experience.

Shinyscreen was designed to fill the niche in-between. Its focus is exclusively on data exploration, visualisation and automated quality control combined with manual overrides to offer simple but powerful functionality without the complexity of the advanced workflows. Unlike other tools that give users unrestricted access to raw MS data—the vast majority of which is irrelevant for a particular investigative effort—the Shinyscreen approach is centered on a preselected list of compounds or masses of interest. Thus, it can be used to support a wide variety of HRMS workflows, including target, suspect (“known”) and NTA (“unknown”) data interrogation without a confusing array of options that can overwhelm inexperienced users and students learning the basics of data processing.

This paper introduces Shinyscreen as a versatile and user-friendly interface for high-throughput HRMS data quality control, discussing the design principles, and demonstrating its utility through real-world applications. By bridging the usability gap between low-level programming tools and complex, resource-intensive platforms, Shinyscreen empowers researchers to focus on their investigative goals, enhancing both accessibility and efficiency in MS data exploration, while also serving a role in teaching activities at the University.

## Implementation

**Fig. 1:**
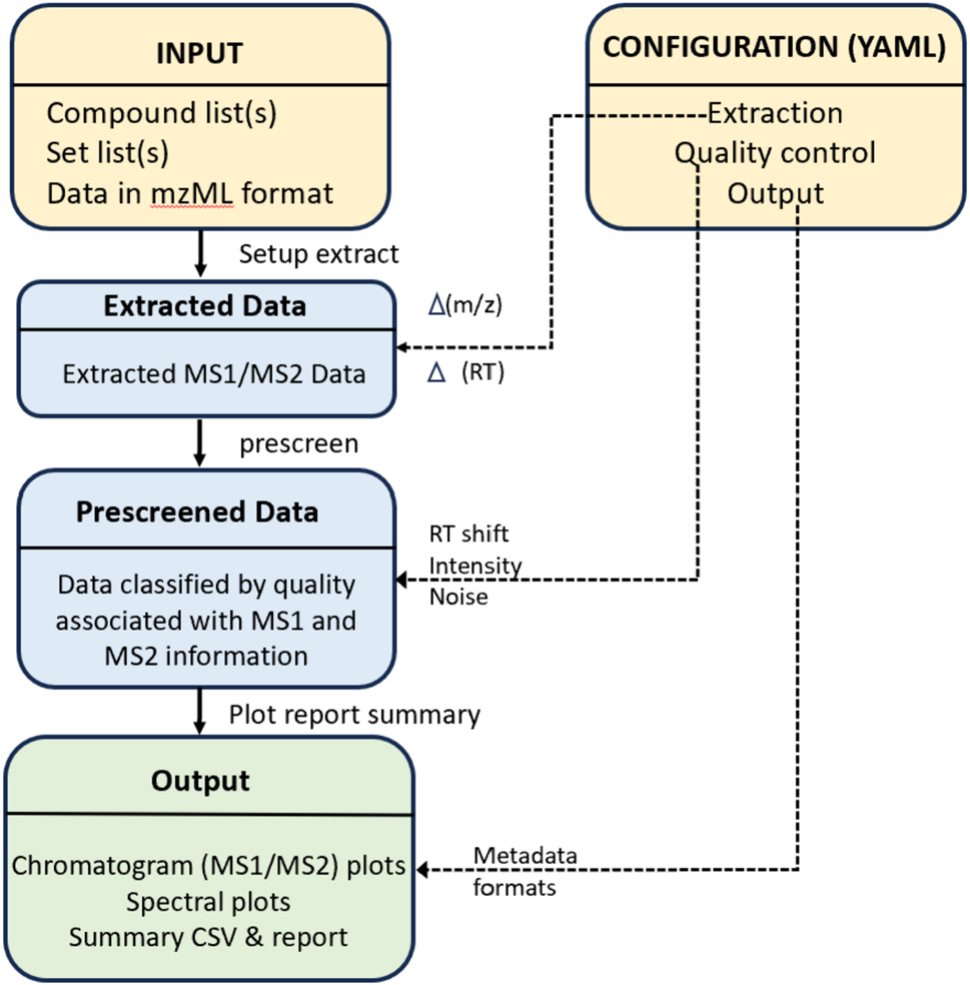
Main stages of Shinyscreen workflow

Shinyscreen is built as a modular [19], extensible architecture that seamlessly integrates user accessibility with computational efficiency. It is tailored to support a wide range of MS data analysis ranging from target and suspect screening workflows to non-targeted studies. The application emphasizes streamlined data processing and intuitive visualisation, making it versatile for different research scenarios and educational purposes. A comprehensive overview of the conceptual workflow for Shinyscreen is presented in Fig. 1, outlining each step of the process. The core steps of Shinyscreen include initialisation, extraction and prescreening, visualisation and integration with MetFrag for further analysis, all wrapped in a front-end (user interface). Following the inputs and configuration (shown in yellow), data is first extracted and then prescreened (blue boxes in Fig. 1) and finally visualized / exported (green box, Fig. 1). This section describes the underlying software framework and technical design of Shinyscreen, while the results and discussion section gives a practical demonstration of Shinyscreen, highlighting its capabilities and key features.

## Software dependencies

Shinyscreen is distributed as an R package. Its source code is primarily written in the R language, with some support code written in JavaScript. Several external dependencies are required for asynchronous graphical interface design, htmltools [20] for customisation of some GUI widgets, withr [21] for safe directory excursions, data.table [22] for efficient implementation of the internal Shinyscreen database, yaml [23] for processing of YAML files and RColorBrewer (RRID:SCR_016697) [24] for plotting.

The application includes various functions to handle the data extraction, prescreening, visualisation and integration with MetFrag. The data.table package is used extensively for handling mass spectrometry data for reading, writing and processing large internal tabular datasets. The DT package [25] integrates interactive tables into shiny interface allowing filtering and dynamic data exploration, thereby improving user engagement and accessibility [14]. The enviPat package (RRID:SCR_003034) [26] provides rapid calculation of adduct masses from molecular formulas, supporting workflows that require precise mass calculations. Finally, ggplot2 [27] serves as the primary tool creating high quality graphical outputs essential for interpreting complex datasets. Complementing ggplot2, the cowplot package [28] is utilized to arrange multiple plots in cohesive layouts within the application. Together, these packages optimize Shinyscreen’s data processing capabilities, ensuring robust, interactive, and visually informative outputs suited to large-scale cheminformatics applications.

## Shinyscreen workflow

### Initialisation

Any Shinyscreen workflow is based on two fundamental groups of inputs: the compound list (in CSV format) and experimental data files (in mzML format). The compound lists contain information describing the compounds of interest. For non-target (unknown) workflows, mass-to-charge (*m/z*) ratios of the precursors of interest are required, as well as, optionally, the expected retention times of the precursors. This input may often come via statistical or other prioritisation methods. For target/suspect (known) workflows, structural data can be provided instead of mass, along with (optional) retention time information.

If some degree of structural information about the compounds is known for example a molecular formula, or a SMILES [29] string as in target or suspect workflows Shinyscreen will automatically calculate required precursor ion masses for extraction, using the given adduct setting. If no structural information is known, the precursor ion masses must be supplied directly. An additional step required for the command-line mode of operation is to parametrise (a) experimental *m/z* and retention time errors; (b) quality control parameters such as MS1 and MS2 intensity thresholds and retention time shifts; and (c) output visualisations and metadata. Default values are provided, based on Orbitrap settings, but will need to be adjusted for other instrumentation.

### Extraction and prescreening

At this stage, the precursor ion masses from the compound lists, their extracted ion chromatograms (EICs, within the specified *m/z* tolerance) as well as their corresponding MS2 spectral scans are retrieved from the data files. The data is extracted from mzML files using the MSnbase package which enables precise retrieval of extracted ion chromatograms (EICs) and MS2 spectra from mzML files. This is the most time-intensive step of the process. The extraction process can be carried out concurrently on multiple files using the future package [30]. This parallelization significantly reduces processing time, particularly for large-scale analyses involving multiple mzML files.

**Fig. 2:**
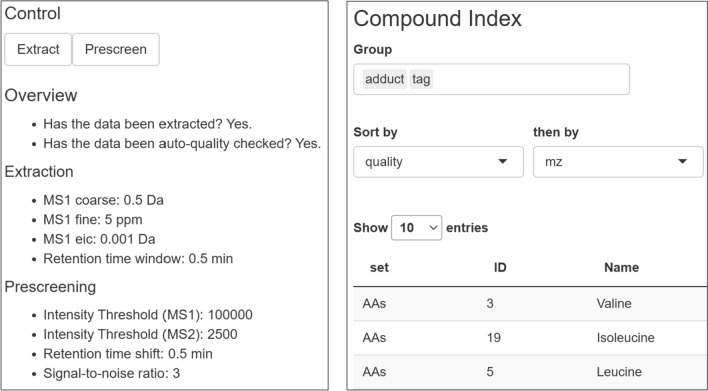
Left: Shinyscreen status overview showing extraction parameters. Right: Compound index after prescreening

**Fig. 3:**
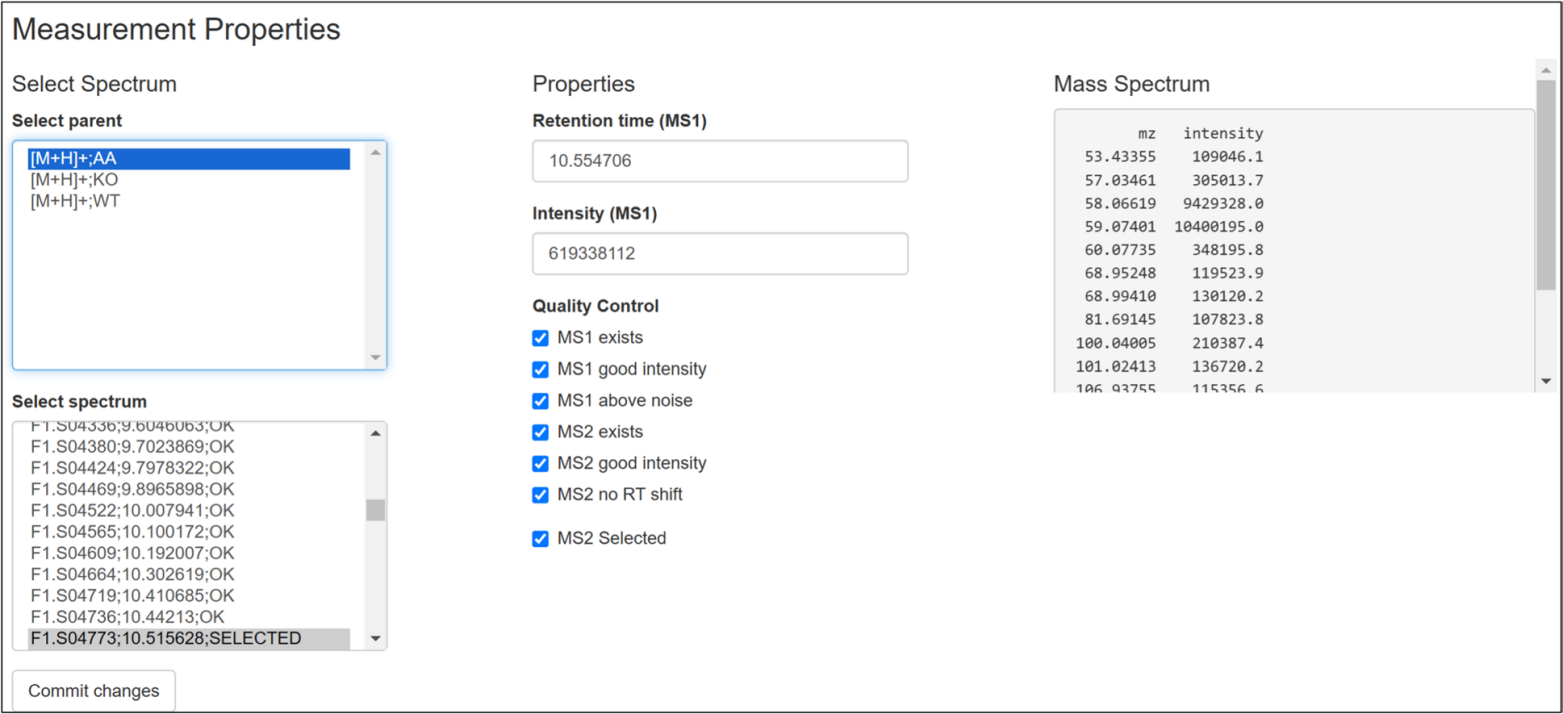
Measurement properties (quality control) pane

After the extraction is done, the data is subjected to a quality assessment stage. The quality of each MS1 parent data point is assessed based on the existence of MS1 and MS2 data, the noise level and the retention time shift compared with the provided thresholds. This step is also used to select a representative MS2 spectra (if present) for the input masses. These checks are all automated based on the input parameters, but can be reviewed manually once processing is done. The GUI will inform the user when the extraction is done and summarise the main parameters (Fig. 2, left). After prescreening, it is possible to navigate the spectra via the Compound Index (Fig. 2, right), using fully searchable tables. It is also possible to filter based on quantities of interest such as *m/z*, retention time, or quality score. A detailed overview of the experimental properties and quality for a particular measurement is available in the Measurement Properties pane (Fig. 3).

### Visualisation

**Fig. 4:**
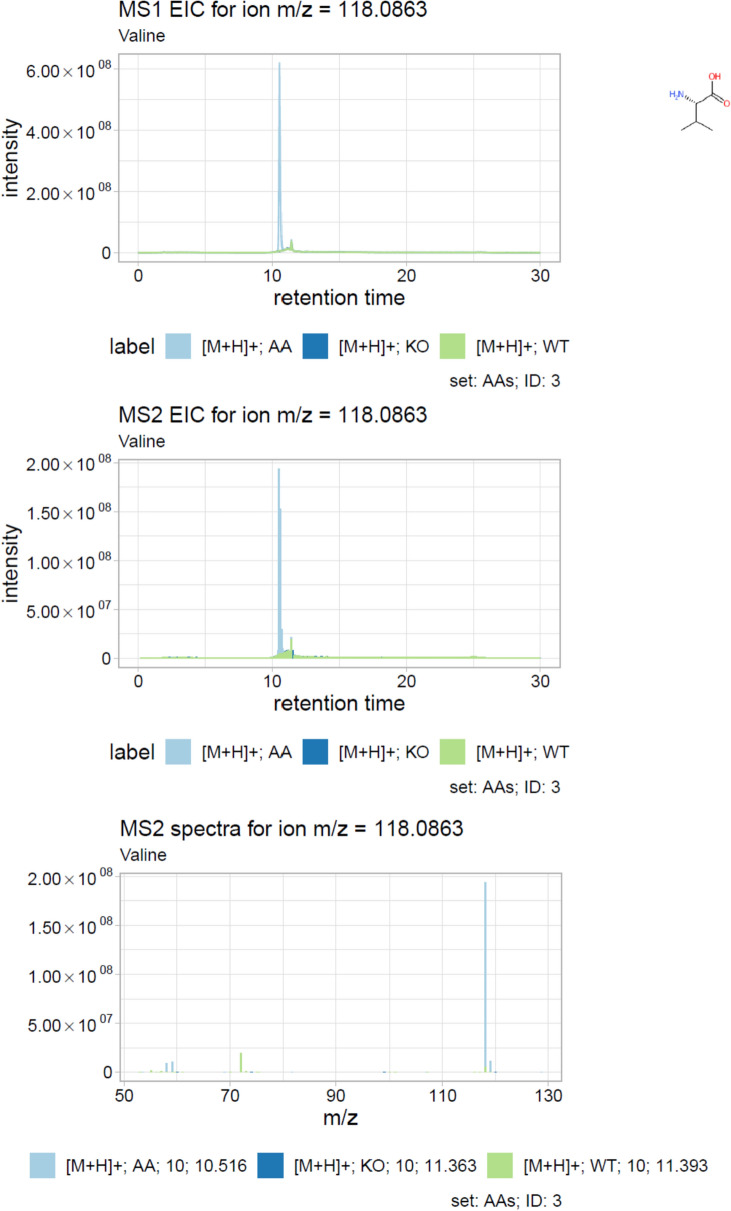
An example plot from Shinyscreen consisting of MS1 chromatogram (top), MS2 chromatogram (middle) and representative MS2 spectrum (bottom) for Valine (structure shown in inset)

Shinyscreen’s visualization capabilities are integral to its user centric design. The ggplot2 [27] and cowplot [28] R packages are employed to generate high-quality plots of MS1 and MS2 chromatograms, as well as MS2 spectra. These visual outputs are designed for both on-screen inspection and publication purposes (see Fig. 4). Shinyscreen also incorporates the DT package to enable dynamic, interactive data exploration through the GUI. Users can sort and query data based on parameters such as *m/z*, retention time, or quality scores thus enhancing efficiency and accessibility.

### Integration with MetFrag

**Fig. 5:**
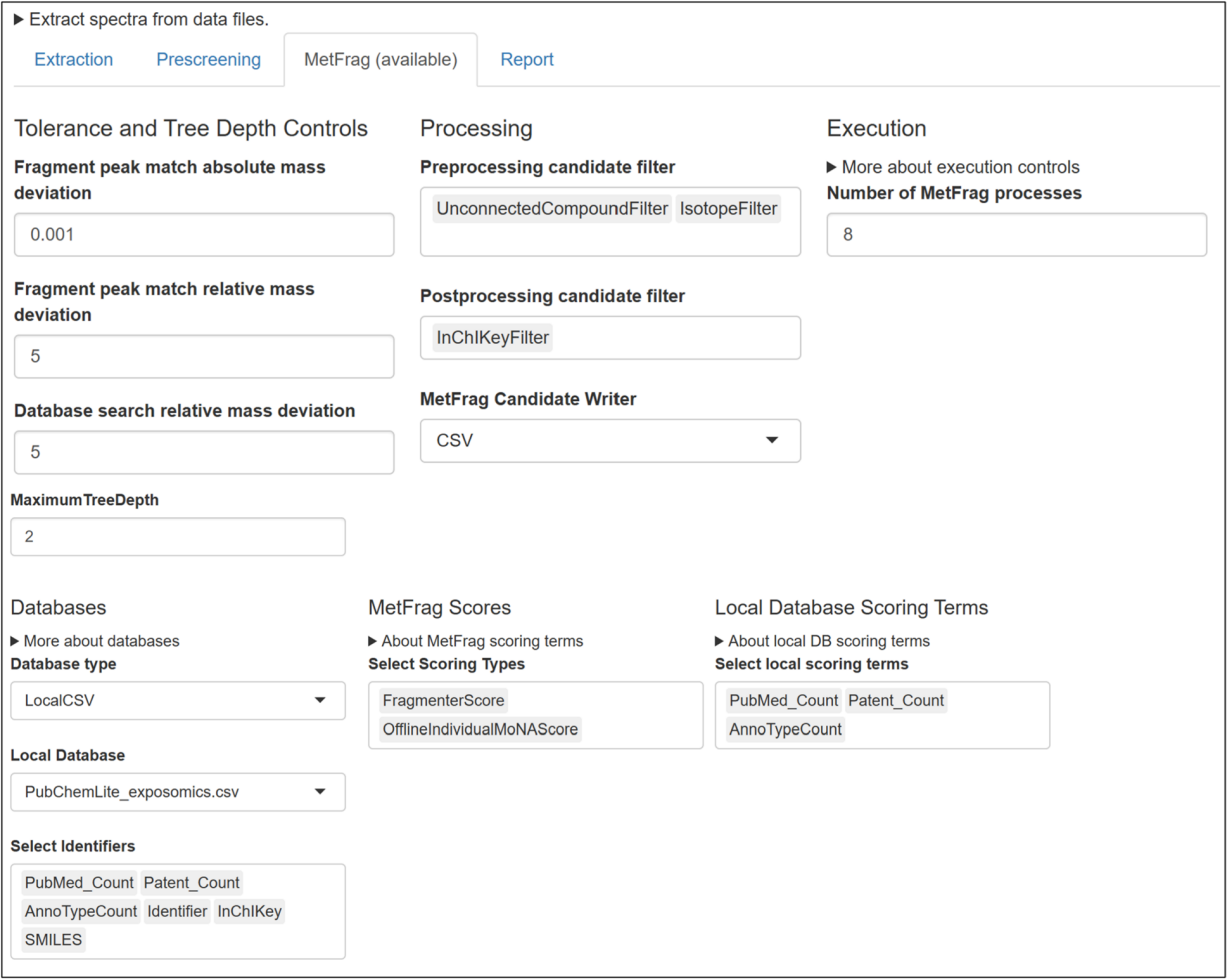
MetFrag Configuration Interface within Shinyscreen

Shinyscreen integrates MetFrag, a widely used tool for in silico fragmentation and candidate structure ranking [15]. A local CSV database version is typically employed to rank candidate structures based on MS2 information, with the current default use set to PubChemLite [31, 32]. While the local runs are much better option for large datasets, the configuration can be complicated for databases with advanced scoring terms such as PubChemLite, and especially for data processing with many MS2 inputs. Shinyscreen provides a MetFrag configuration interface (Fig. 5) that hides a lot of the complexity and runs several MetFrag instances in background to speed up the analysis. The integration is streamlined through an automated configuration interface, hiding the complexity of MetFrag setup. Shinyscreen has been primarily tested with local databases, such as PubChemLite, which is downloaded from Zenodo (RRID:SCR_004129) within the Docker image. PubChemLite includes additional fields, such as annotation count, patent count, and PubMed (RRID:SCR_004846) count, which can be utilized by MetFrag as additional scoring terms to aid in the ranking of candidate structures. The results are aggregated into summary tables, allowing users to efficiently review candidate structures and their associated scores for all the MS2 spectra that passed the quality control criteria.

### Output

Shinyscreen provides a range of different outputs. There are plots of MS1 and MS2 chromatograms coupled with MS2 spectra organised in a PDF report (Fig. 4), providing visual summaries. Then, there are global summaries in CSV format. These include (a) a summary table consolidating key metadata, quality control results, spectral and experimental information such as retention times and peak intensities; (b) an MS2 spectra table, containing the precursor information for all MS2 spectra, facilitating further analysis and interpretation, and (c) the MetFrag summary table summarising MetFrag candidates for extracted MS2 spectra. Finally, the object returned by a prescreen call contains the extracted data, associations between MS1 and MS2, as well as other data generated by Shinyscreen during the execution of the pipeline organised in multiple related tables. This is the lowest level of all the outputs, but offers the largest degree of flexibility in terms of further processing.

## Extensibility, deployment and scalability

To combat installation challenges in windows and mac platforms (since the primary development operating system (OS) was Linux), a Docker image was created using a Linux OS base image to encapsulate Shinyscreen along with its dependencies. This allowed Shinyscreen to run seamlessly on Windows through Docker Desktop, circumventing the compatibility issues and enabling a consistent, reliable environment for the users. The Docker image, which includes all necessary dependencies and configurations, is now available in the container registry [33]. This approach not only resolved the installation and execution issues but also enhances the portability of the software, making it more accessible to a broader range of users across different operating systems. This solution also paved the way for the next phase of the project’s deployment, where the Docker container can be easily integrated into a variety of platforms and computing environments, including cloud-based systems, local servers, and multi-user configurations, while also optimizing the deployment and scalability of Shinyscreen.

In Shinyscreen’s deployment, a Continuous Integration (CI) pipeline is configured within GitLab to support automated testing and streamline application updates. The CI pipeline runs builds and tests to provide with immediate feedback on the code integrity. This pipeline also simplifies the creation of installation files, which bundle all dependencies required for deploying Shinyscreen effectively. For multi-user educational access, Shinyscreen is hosted within a Kubernetes cluster, designed to support scalability and resource management for a teaching platform (ECI-ISB401). The Kubernetes setup allows multiple students to use the application simultaneously, with dynamic allocation of computational resources to optimize performance and user experience. Together, the CI/CD pipeline, Docker containerization, and Kubernetes orchestration create a robust, accessible, and scalable platform tailored to both individual and multi-user settings.

## Results and discussion

### Front-end (user interface)

**Fig. 6:**
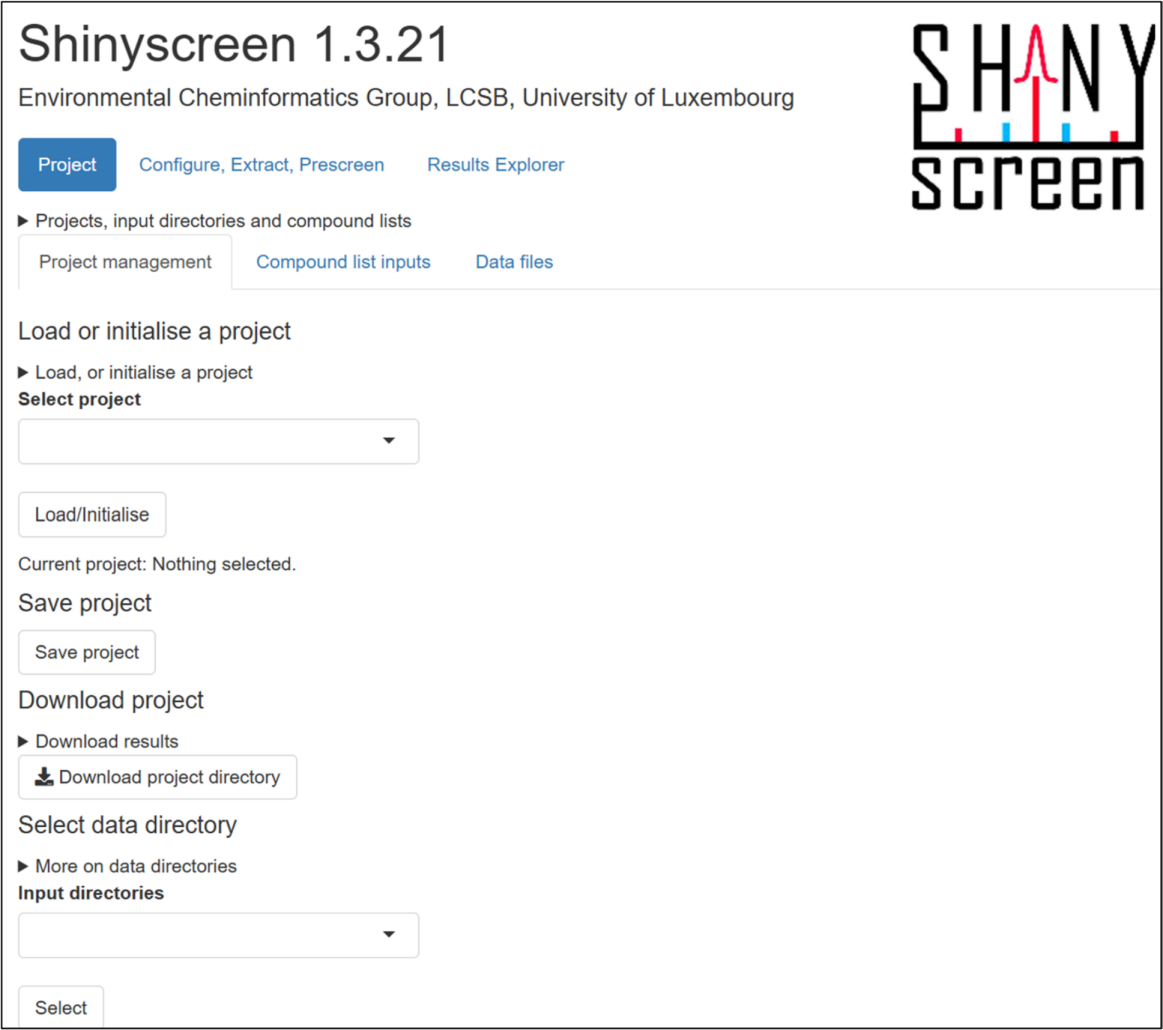
Shinyscreen user interface

Shinyscreen is developed using the R Shiny framework with an intent to support web-based data analysis and visualisation. The user interface (UI) for Shinyscreen (Fig. 6) is defined in Shiny UI scripts using fluid layouts to enable data input, visualisation and control elements. Each UI component is reactive, automatically updating in response to user interactions, such as modifying input configurations or adjusting analysis parameters. This design ensures an interactive and intuitive user experience, enabling efficient handling of complex mass spectrometry data in real-time.

**Fig. 7:**
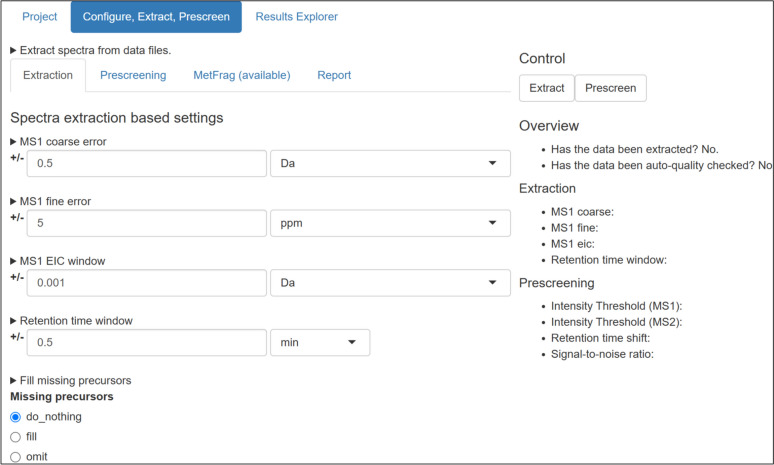
Shinyscreen configuration screen

Configuration can also be done using the GUI as in Fig. 7.

Another feature of Shinyscreen is interactivity. A great deal of development effort has been poured into a self-documented web-based interface which enacts a seamless configuration—extraction—prescreening—visualisation loop (see Fig. 1). Moreover, it is possible to serve the web interface in a multi-user environment, such as a lab computing platform, or workshops. Finally, Shinyscreen has been designed to fit into more comprehensive workflows. It can run without GUI, in headless mode, on local computers, servers and clusters. Shinyscreen outputs are self-contained and available in interchangeable formats such as RDS and CSV. Not only the data, but also the program itself is embeddable by the virtue of a top-level R API.

While Shinyscreen currently leverages Shiny’s reactive framework, the GUI could potentially be enhanced in the future by decoupling the front end from Shiny’s default rendering mechanisms. This would enable the integration of modern JavaScript-based frameworks, such as React or Vue.js, for a more dynamic, scalable, and user-friendly interface, while maintaining the robust analytical backend in R.

## Environmental studies

Shinyscreen has been applied in several studies. Shinyscreen was used in a study investigating pesticides and their transformation products (TPs) in Luxembourgish waters [18]. By enabling targeted and non-targeted analysis of HRMS data, Shinyscreen facilitated the discovery and characterization of contaminants, showcasing its utility in environmental cheminformatics workflows. Similarly, it was utilized to study the occurrence and distribution of pharmaceuticals and their TPs in surface waters [34], highlighting its capacity for processing complex environmental datasets and supporting public health research. In addition, Shinyscreen has been used in adding open spectral data (5546 records) to public databases with high-quality mass spectra [3]. This includes contributions to resources such as MassBank and PubChem, enabling improved compound annotation for non-targeted exposomics of chemical mixtures. These efforts support global initiatives for open data sharing and enhance the reproducibility of non-targeted analytical workflows.

## Teaching

Since 2020, Shinyscreen has been an integral part of the master’s teaching curriculum ISB401 at the University of Luxembourg, where it is used to familiarize students with MS data analysis. The teaching module is centered on targeted and non-targeted analysis of amino acids in *Saccharomyces cerevisiae* (yeast) wild-type and knock-out strains. This data set also serves as the test data provided in the package vignette. The practical session not only provides students with experience in handling complex HRMS datasets but also introduces them to the principles of data exploration, quality control, and annotation. The output of Shinyscreen can be readily used with MetFragWeb (good for students to learn the features and understand the results during teaching) [15, 35]), or integrated with MetFrag Command Line itself (better for batch processing) [36] for further analysis, enabling seamless integration into workflows for compound annotation. Notably, Shinyscreen’s development was shaped, in part, by feedback and input from the 2019 student cohort, ensuring its relevance as an educational tool both for teaching the subject but also in teaching software development for a non-expert audience. Student feedback has highlighted Shinyscreen as a user-friendly tool that bridges the gap between theoretical knowledge and practical application in MS data analysis while developing a strong foundational understanding of HRMS workflows. These case studies underscore Shinyscreen’s flexibility and robustness, making it a valuable tool for advancing research in environmental chemistry, database curation, and education.

## Conclusion

In conclusion, Shinyscreen’s prescreening capabilities have proven valuable in different environmental studies, particularly where it has significantly enhanced data analysis and compound annotation. Shinyscreen has been especially beneficial for students, providing them with a practical application in MS studies while also fostering a deeper understanding of open source software development. The open source nature of Shinyscreen has encouraged collaboration, facilitating its use and continuous improvement within the research community. Furthermore, ongoing collaboration with the patRoon team aims to make both tools complementary in mass spectrometry workflows, further enhancing research capabilities. We welcome feedback and new use cases to continue refining Shinyscreen’s functionality.

## Availability and requirements

- **Project name**: Shinyscreen
- **Project home page**: https://gitlab.com/uniluxembourg/lcsb/eci/shinyscreen
- **Operating system(s)**: Platform independent
- **Programming language**: R (93%), JavaScript (4.5%)
- **Other requirements**: R 4.0.0 or higher
- **License**: Apache 2.0
- **Any restrictions to use by non-academics**: None

## Data Availability

The Shinyscreen code is available on the ECI GitLab (https://gitlab.com/uniluxembourg/lcsb/eci/shinyscreen) under the Apache License 2.0, and the corresponding Docker image can be accessed via the container registry [33]. All resources and documentation are provided under open licenses, ensuring accessibility and reproducibility. No datasets were generated or analysed during the current study.

## References

[1] Kuril AK (2024) Exploring the versatility of mass spectrometry: applications across diverse scientific disciplines. Eur J Mass Spectrom 30:209–220. 10.1177/14690667241278110. (**[cito:citesAsAuthority]**) 10.1177/14690667241278110 39314187

[2] Hollender J, Schymanski EL, Ahrens L et al (2023) NORMAN guidance on suspect and non-target screening in environmental monitoring. Environ Sci Eur 35:75. 10.1186/s12302-023-00779-4. (**[cito:citesAsAuthority]**)

[3] Elapavalore A, Kondić T, Singh RR et al (2023) Adding open spectral data to MassBank and PubChem using open source tools to support non-targeted exposomics of mixtures. Environ Sci Process Impacts 25:1788–1801. 10.1039/D3EM00181D. (**[cito:citesAsAuthority]**) 37431591 10.1039/d3em00181d PMC10648001

[4] Matthiesen R, Bunkenborg J (2020) Introduction to mass spectrometry-based proteomics. In: Matthiesen R (ed) Mass spectrometry data analysis in proteomics. Springer, New York, NY, pp 1–58 (**[cito:citesAsAuthority]**)

[5] Thermo Scientific (2024) Xcalibur™ Software. https://www.thermofisher.com/order/catalog/product/OPTON-30967. Accessed 2 Dec 2024

[6] Wenig P, Odermatt J (2010) OpenChrom: a cross-platform open source software for the mass spectrometric analysis of chromatographic data. BMC Bioinformatics 11:405. 10.1186/1471-2105-11-405. (**[cito:citesAsAuthority]**) 20673335 10.1186/1471-2105-11-405 PMC2920884

[7] Sturm M, Bertsch A, Gröpl C et al (2008) OpenMS—an open-source software framework for mass spectrometry. BMC Bioinformatics 9:163. 10.1186/1471-2105-9-163. (**[cito:citesAsAuthority]**) 18366760 10.1186/1471-2105-9-163 PMC2311306

[8] Petras D, Phelan VV, Acharya D et al (2022) GNPS dashboard: collaborative exploration of mass spectrometry data in the web browser. Nat Methods 19:134–136. 10.1038/s41592-021-01339-5. (**[cito:citesAsAuthority]**) 34862502 10.1038/s41592-021-01339-5 PMC8831450

[9] Fischer B, Neumann S, Gatto L, Kou Q (2017) mzR: parser for netCDF, mzXML, mzData and mzML and mzIdentML files (mass spectrometry data). http://bioconductor.org/packages/mzR/. Accessed 2 Dec 2024 [cito:citesAsAuthority]

[10] Gatto L, Gibb S, Rainer J (2021) MSnbase, efficient and elegant R-based processing and visualization of raw mass spectrometry data. J Proteome Res 20:1063–1069. 10.1021/acs.jproteome.0c00313. (**[cito:citesAsAuthority]**) 32902283 10.1021/acs.jproteome.0c00313

[11] Laurent Gatto JR (2017) MSnbase. https://bioconductor.org/packages/MSnbase. Accessed 24 May 2025 [cito:citesAsAuthority]

[12] Colin A. Smith <Csmith@Scripps. Edu> RTC (2024) Xcms: LC-MS and GC-MS Data Analysis. https://bioconductor.org/packages/xcms. Accessed 6 Dec 2024 [cito:citesAsAuthority]

[13] Gatto L, Gibb S, Rainer J (2024) R for mass spectrometry. https://www.rformassspectrometry.org/. Accessed 6 Dec 2024

[14] Helmus R, ter Laak TL, van Wezel AP et al (2021) patRoon: open source software platform for environmental mass spectrometry based non-target screening. J Cheminform 13:1. 10.1186/s13321-020-00477-w. (**[cito:citesAsAuthority]**) 33407901 10.1186/s13321-020-00477-w PMC7789171

[15] Ruttkies C, Schymanski EL, Wolf S et al (2016) MetFrag relaunched: Incorporating strategies beyond in silico fragmentation. J Cheminform 8:3. 10.1186/s13321-016-0115-9. (**[cito:citesAsAuthority]**) 26834843 10.1186/s13321-016-0115-9 PMC4732001

[16] Stravs M, Schymanski E, Neumann S, et al (2020) RMassBank: workflow to process tandem MS files and build MassBank records. https://bioconductor.org/packages/RMassBank/. Accessed 20 Jan 2020 [cito:citesAsAuthority]

[17] Stravs MA, Schymanski EL, Singer HP, Hollender J (2013) Automatic recalibration and processing of tandem mass spectra using formula annotation: recalibration and processing of MS/MS spectra. J Mass Spectrom 48:89–99. 10.1002/jms.3131. (**[cito:citesAsAuthority]**) 23303751 10.1002/jms.3131

[18] Krier J, Singh RR, Kondić T et al (2022) Discovering pesticides and their TPs in Luxembourg waters using open cheminformatics approaches. Environ Int 158:106885. 10.1016/j.envint.2021.106885. (**[cito:citesAsAuthority]**) 34560325 10.1016/j.envint.2021.106885 PMC8688306

[19] Wikipedia (2025) Modular programming. In: Wikipedia. https://en.wikipedia.org/wiki/Modular_programming. Accessed 24 May 2025

[20] Cheng J, Sievert C, Schloerke B, et al (2024) Htmltools: tools for HTML. https://cran.r-project.org/web/packages/htmltools/index.html. Accessed 18 Nov 2024 [cito:citesAsAuthority]

[21] Hester J, Henry L, Müller K, et al (2024) Withr: run code ‘With’ temporarily modified global state. https://cran.r-project.org/web/packages/withr/index.html. Accessed 18 Nov 2024 [cito:citesAsAuthority]

[22] Barrett T, Dowle M, Srinivasan A, et al (2024) Data.table: extension of ’data.frame’. https://cran.r-project.org/web/packages/data.table/index.html. Accessed 18 Nov 2024 [cito:citesAsAuthority]

[23] Garbett SP, Stephens J, Simonov K, et al (2024) Yaml: methods to convert R data to YAML and back. https://cran.r-project.org/web/packages/yaml/index.html. Accessed 18 Nov 2024 [cito:citesAsAuthority]

[24] Neuwirth E (2022) RColorBrewer: ColorBrewer Palettes. https://cran.r-project.org/web/packages/RColorBrewer/index.html. Accessed 17 Nov 2024 [cito:citesAsAuthority]

[25] Xie Y, Cheng J, Tan X, et al (2024) DT: a wrapper of the JavaScript library ’DataTables’. https://cran.r-project.org/web/packages/DT/index.html. Accessed 18 Nov 2024 [cito:citesAsAuthority]

[26] Loos M, Gerber C, Corona F et al (2015) Accelerated isotope fine structure calculation using pruned transition trees. Anal Chem 87:5738–5744. 10.1021/acs.analchem.5b00941. (**[cito:citesAsAuthority]**) 25929282 10.1021/acs.analchem.5b00941

[27] Wickham H (2016) ggplot2. http://link.springer.com/10.1007/978-3-319-24277-4. Accessed 13 Nov 2024 [cito:citesAsAuthority]

[28] Wilke CO (2024) Cowplot: streamlined plot theme and plot annotations for ’ggplot2’. https://cran.r-project.org/web/packages/cowplot/index.html. Accessed 18 Nov 2024 [cito:citesAsAuthority]

[29] Weininger D (1988) SMILES, a chemical language and information system. 1. introduction to methodology and encoding rules. J Chem Inf Comput Sci 28:31–36. 10.1021/ci00057a005. (**[cito:citesAsAuthority]**)

[30] Bengtsson H (2021) A unifying framework for parallel and distributed processing in R using futures. R J 13:208. 10.32614/RJ-2021-048. (**[cito:citesAsAuthority]**)

[31] Schymanski EL, Kondić T, Neumann S et al (2021) Empowering large chemical knowledge bases for exposomics: PubChemLite meets MetFrag. J Cheminform 13:19. 10.1186/s13321-021-00489-0. (**[cito:citesAsAuthority]**) 33685519 10.1186/s13321-021-00489-0 PMC7938590

[32] Elapavalore A, Ross DH, Grouès V et al (2025) PubChemLite plus collision cross section (CCS) values for enhanced interpretation of nontarget environmental data. Environ Sci Technol Lett. 10.1021/acs.estlett.4c01003. (**[cito:citesAsAuthority]**) 39957787 10.1021/acs.estlett.4c01003 PMC11823450

[33] Elapavalore A (2025) Shinyscreen container registry. In: GitLab. https://gitlab.com/uniluxembourg/lcsb/eci/shinyscreen/container_registry. Accessed 19 Mar 2025 [cito:citesAsAuthority]

[34] Singh RR, Lai A, Krier J et al (2021) Occurrence and distribution of pharmaceuticals and their transformation products in luxembourgish surface waters. ACS Environ Au 1:58–70. 10.1021/acsenvironau.1c00008. (**[cito:citesAsAuthority]**) 37101936 10.1021/acsenvironau.1c00008 PMC10114791

[35] IPB Halle (2024) MetFrag web. https://msbi.ipb-halle.de/MetFrag/. Accessed 3 Dec 2024

[36] IPB Halle (2024) MetFrag command line (CL). http://ipb-halle.github.io/MetFrag/projects/metfragcl/. Accessed 3 Dec 2024

